# Protective effects of oxymatrine against DSS-induced acute intestinal inflammation in mice via blocking the RhoA/ROCK signaling pathway

**DOI:** 10.1042/BSR20182297

**Published:** 2019-07-19

**Authors:** Yifan Wang, Zhexing Shou, Heng Fan, Meng Xu, Qianyun Chen, Qing Tang, Xingxing Liu, Hui Wu, Man Zhang, Ting Yu, Shuangjiao Deng, Yujin Liu

**Affiliations:** Department of Integrated Traditional Chinese and Western Medicine, Union Hospital, Tongji Medical College, Huazhong University of Science and Technology, Wuhan 430022, China

**Keywords:** immunity, intestinal epithelial barrier, oxidative stress, Oxymatrine, RhoA/ROCK pathway, ulcerative colitis

## Abstract

Oxymatrine (OMT) is an important quinoxaline alkaloid that has a wide range of pharmacological effects and has been shown to alleviate ulcerative colitis due to its profound anti-inflammatory effects. The RhoA/ROCK (Rho kinase) signaling pathway has been shown to be related to the pathogenesis of several autoimmune diseases; however, the specific mechanisms of RhoA/ROCK signaling in inflammatory bowel disease (IBD) remain elusive. Therefore, we sought to determine whether OMT could ameliorate acute intestinal inflammation by targeting the RhoA/ROCK signaling pathway. The potential therapeutic effect of OMT on acute intestinal inflammation and its impact on the RhoA/ROCK signaling pathway were assessed in six groups of mice treated with low, medium and high doses of OMT (25, 50 and 100 mg/kg, respectively), and an inhibitor of ROCK, Y-27632, as a positive control, after initiating dextran sodium sulfate (DSS)-induced acute intestinal inflammation. The model group and normal group were injected intraperitoneally with equal doses of PBS. Our results showed that OMT treatment could protect the integrity of the epithelial barrier, relieve oxidative stress, inhibit the expression of inflammatory mediators and pro-inflammatory cytokines, restrain the differentiation of Th17 cells and promote the differentiation of Treg cells via inhibition of the RhoA/ROCK pathway, thus providing therapeutic benefits for ulcerative colitis (UC). Therefore, inhibiting the RhoA/ROCK pathway might be a new approach that can be used in UC therapy, which deserves to be investigated further.

## Introduction

Inflammatory bowel disease (IBD) is a class of chronic intestinal diseases that primarily includes ulcerative colitis (UC) and Crohn’s disease (CD) [1]. Although the etiology and pathology of IBD remain elusive, several factors have been found to play pivotal roles in both the development and incidence of IBD, including genetic factors, immune dysfunction, nitrosative and oxidative stress, intestinal epithelial barrier (IEB) dysfunction and environmental factors [2,3]. The clinical manifestations of UC include abdominal pain, diarrhea and mucosanguineous feces, which are closely related to the degree and severity of inflammation [4]. Currently available treatments are effective and beneficial for limiting disease progression. However, there is little chance of recovery if these treatments are used in isolation. Current treatment methods include immunosuppressive drugs, amino salicylates and glucocorticoids, which are mainly used to treat IBD [5–7]. Unfortunately, the side effects of these treatments have limited their use [7,8]. Therefore, in addition to conventional treatment, there is an urgent need to use herbal medicines as alternative and adjuvant treatments [9,10].

Oxymatrine (OMT) is a natural quinoxaline alkaloid and the primary biologically active ingredient extracted from the root of the Chinese herbal medicine *Sophora flavescens* Ait. OMT has a variety of pharmacological effects, including anti-inflammatory effects [11], proapoptotic effects [12], antioxidative effects [13], immunomodulatory effects [14], antiviral effects [15], antitumor effects [16], antiproliferative effects [17], anti-allergy effects, antifibrotic effects and cardiovascular protective effects [18]. Recently, extensive experimental studies of OMT have been performed to expand its use in UC therapy, largely because of its anti-inflammatory properties [11,19].

Cdc42, Rac and RhoA, which are recognized as small GTPases of the Rho family, are involved in many cellular processes, including cell structure, reactive oxygen species (ROS) formation, cell adhesion and migration, apoptosis, actin cytoskeletal movements and cell differentiation [20]. Rho kinase (ROCK) is a 160 kDa threonine/serine kinase made up of ROCK2 and ROCK1. ROCK1 and ROCK2 were initially identified as key effectors of RhoA, and through the phosphorylation of the downstream targets, these kinases regulate a wide range of physiological functions [21]. ROCK has many types of downstream targets, including myosin light chain (MLC), MLC phosphatase (MLCP) and myosin phosphatase-targeting subunit-1 (MYPT-1). Moreover, NF-κB, an important transcription factor, is another downstream target of ROCK and plays a significant role in the pathogenesis of IBD. Both the expression and activation of NF-κB are strongly induced in macrophages and intestinal epithelial cells isolated from inflamed tissues of patients with IBD. Furthermore, there is a strong relationship between the degree of NF-κB activation and the severity of colonic inflammation [22,23]. Recently, the RhoA/ROCK signaling pathway has been proved to be associated with the level of immune system activation and the production of pro-inflammatory factors [24]. Horowitz et al. has demonstrated that significantly higher AR activity and increased RhoA activity can be observed in the submucosal tissues of IBD around the microvessels compared with the normal control intestinal tissues [25]. Epithelial permeability is primarily controlled by tight junctions (TJs), while stable cell–cell adhesion is dependent on adherens junctions (AJs). AJs and TJs together form the apical junction complex that controls epithelial barrier integrity. It has also been demonstrated that actin contractile force of RhoA/rock1 dependent actomyosin was increased due to cortactin deficiency and changes in adherens junctions (AJs) and TJs molecular composition resulted in increased permeability. This barrier defect was not enough to induce spontaneous colitis, but it significant aggravated experimental colitis in cortactin- Ko mice [26].

Considering the crucial role of RhoA/ROCK signaling pathway and the therapeutic effect of OMT in UC, the present study was to investigate whether OMT could alleviate DSS-induced acute intestinal inflammation by protecting the integrity of the epithelial barrier, relieving oxidative stress, inhibiting inflammation and restoring Th17/Treg cell balance through targeted down-regulation of the RhoA/ROCK signaling pathway.

## Materials and methods

### Animals

Male, specific-pathogen-free (SPF) Balb/c mice (body weight: 18–22 g) were provided by the Laboratory Animal Center of the Huazhong University of Science and Technology (HUST, Wuhan, China) (Quality Certification of Laboratory Animals: SCXK(e)2016-0009). Each mouse was maintained under a controlled temperature (22°C) and photoperiod in the Laboratory Animal Center of the HUST (Permit number:SYXK(e)2016-0057). All experimental procedures strictly adhered to the guidelines established by the Animal Research Committee of HUST. Every animal experiment was evaluated and approved by the Institutional Animal Care and Use Committee (IACUC) of HUST.

### Experimental design

Through oral administration of 3.0% DSS (36–50 kDa; MP Biomedicals, CA, U.S.A.) in fresh drinking water for 1 week, we generated an acute intestinal inflammation animal model in Balb/c mice (*n*=8). Starting on the first day of model induction, intraperitoneal injection of 25 mg/kg, 50 mg/kg or 100 mg/kg of OMT (Meilunbio, Dalian, China) [27] and 10 mg/kg of ROCK inhibitor, Y-27632 (Tocris Bioscience, Bristol, U.K.) was performed in the treatment group for 7 days. The model group and the normal group were injected with equal doses of PBS intraperitoneally.

### Assessment of DSS-induced intestinal inflammation

To assess the disease activity index (DAI), fecal occult blood, fecal consistency and body weight were observed every day during all DSS treatments. All groups were anesthetized and euthanized by cervical dislocation. To perform histological analyses, the colons were dissected as previously described [28].

### Western blot analysis

Western blot analysis was used to quantify the expression levels of proteins in colonic tissues as previously described [28]. RIPA lysis buffer mixed with protease inhibitor cocktail was used to extract the total protein (Roche, Basel, Switzerland). After that, the protein concentration was measured by a BCA protein assay kit (ASPEN, Wuhan, China) based on the manufacturer’s protocol. Sodium dodecyl sulfate-polyacrylamide gel electrophoresis was used to separate proteins. Then, the protein was transferred onto a polyvinylidene difluoride membrane, and 5% nonfat skim milk was used to block the membranes, which were then incubated overnight at 4°C with appropriate primary antibodies against β-actin (1:10,000; TDY Biotech, Beijing, China), ROCK-2 (1:2000; Abcam, Cambridge, U.K.), ROCK-1 (1:2000; Abcam), Occludin (1:2000; Abcam), zonula occluden (ZO)-1 (1:500; Abcam,), Foxp3 (1:1000, Abcam), RORγt (1:500; Biorbyt, Wuhan, China), NF-κBp65 (1:2000; Cell Signaling Technology, Danvers, MA, U.S.A.), p-MYPT-1(Thr696) (1:500; Cell Signaling Technology), p-MLC(Ser19) (1:1000; Cell Signaling Technology), MYPT-1 (1:1000; Cell Signaling Technology), MLC (1:1000; Cell Signaling Technology). Then, TBST was used to wash the membrane, and a secondary antibody conjugated with peroxidase was incubated with the membrane for 1 h. Finally, we visualized and analyzed the protein bands of interest. To make comparisons, we used β-actin expression as a control.

### Immunohistochemistry and immunofluorescence staining

A standard IHC procedure was performed [28], in which 4% paraformaldehyde was used to fix fresh colon tissue samples from mice. Then, 5-µm-thick sections were embedded in paraffin. The sections were then incubated with anti-ZO-1 (1:100; Santa Cruz, CA, U.S.A.), anti-occludin (1:200; Abcam), anti-iNOS (1:50; Abcam) and anti-COX-2 (1:200; Abcam) primary antibodies overnight at 4°C, followed by incubation with a biotinylated secondary antibody. Diaminobenzidine (DAB) was used for color development of sections, along with counterstaining with hematoxylin. We used a light microscope to image the IHC slides. For immunofluorescence staining, we used PBS containing 3% bovine serum albumin (BSA) to pre-incubate dewaxed sections for half an hour. Then, we used 4′,6-diamidino-2-phenylindole (DAPI) to counterstain these sections, and after that, we washed and mounted these sections in antifade medium. A confocal laser scanning microscope (Olympus-FV1000, Tokyo, Japan) was applied to examine the expression levels of cluster of differentiation myeloperoxidase (MPO).

### Quantitative real-time PCR

TRIzol reagent was used (TaKaRa Bio, Inc., Shiga, Japan) to extract total RNA from colonic tissues. Using PrimeScript™ RT Master Mix (TaKaRa), we reverse transcribed the isolated RNAs into cDNA. Subsequently, quantitative real-time PCR (qRT-PCR) was performed to evaluate the expression levels of ROCK-2, ROCK-1, IL-10, IL-2, IL-21, IL-17A, Foxp3, RORγt, iNOS and COX-2 using SYBR Premix Ex Taq™ (TaKaRa). Table 1 shows all utilized primer sequences.

**Table 1:** Primers used for qRT-PCR

Gene		Primer sequences (5′-3′)	Length (bp)
β-Actin	Forward	CTGAGAGGGAAATCGTGCGT	208
	Reverse	CCACAGGATTCCATACCCAAGA	
iNOS	Forward	CATTCAGATCCCGAAACGCT	316
	Reverse	TGTAGGACAATCCACAACTCGC	
COX-2	Forward	AGAGGTGTATCCCCCCACAG	167
	Reverse	TGTCGCACACTCTGTTGTGC	
RORγt	Forward	TGTTTTTCTGAGGATGAGATTGC	161
	Reverse	GCTAGGAGGCCTTGTCGATG	
IL-17A	Forward	CTCAGACTACCTCAACCGTTCC	141
	Reverse	ATGTGGTGGTCCAGCTTTCC	
IL-21	Forward	CATAAATCAAGCCCCCAAGG	197
	Reverse	CCAGGGTTTGATGGCTTGAG	
ROCK-1	Forward	GGACGAGAGTGTGACTGGTGG	219
	Reverse	ACCATTTCTGCCCAATCTCAC	
ROCK-2	Forward	CAGCAACTTTGACGACATTGAG	274
	Reverse	AGATTTGCACTTCTGTTCCAGC	
FOXP3	Forward	ACCACCTTCTGCTGCCACTG	154
	Reverse	AAGGTTGCTGTCTTTCCTGGG	
IL-10	Forward	TACAGCCGGGAAGACAATAACT	142
	Reverse	AGGAGTCGGTTAGCAGTATGTTG	
IL-2	Forward	ATGAACTTGGACCTCTGCGG	222
	Reverse	GAGGGCTTGTTGAGATGATGC	

### Enzyme-linked immunosorbent assay

Enzyme-linked immunosorbent assay (ELISA) kits (ELK Biotechnology, Wuhan, China) were used to measure the levels of IL-17A, IL-2, IL-10 and IL-21 in colon homogenate supernatants via the quantitative sandwich enzyme immunoassay technique based on the manufacturer’s protocol.

### Flow cytometry

Monocytes were obtained from spleens and mesenteric lymph nodes (MLNs) and subjected to intracellular staining to measure the proportions of CD4^+^IL-17A^+^T cells and CD4^+^CD25^+^FOXP3^+^T cells [29]. For the analysis of Th17 cells, the cells were treated with a leukocyte activation cocktail with BD GolgiPlug™ (BD Biosciences, New York, U.S.A.) under an atmosphere of 5% carbon dioxide at 37°C for 6 h. The cultured cells were then stained for surface markers with a FITC-conjugated anti-CD4 antibody (BD Biosciences, New York, U.S.A.) and incubated for 15 min at RT in the dark. The cells were next fixed and permeabilized for 20 min at RT in the dark, followed by staining with a PE-conjugated anti-interleukin-17A antibody (BD Biosciences). For Treg cell analysis, the cells were stained with FITC-conjugated anti-CD4 and PerCP-Cy5.5-conjugated anti-CD25 antibodies (BD Biosciences) for 30 min at 4°C in the dark. The cells were then fixed and permeabilized with fixation/permeabilization working solution and permeabilization buffer for 30 min in the dark and subsequently stained with an AF647-conjugated anti-FOXP3 antibody in the dark. The stained cells were washed with permeabilization buffer (BD Biosciences), followed by analysis via flow cytometry.

### Biochemical analysis

NO, glutathione (GSH), MPO (Nanjing Jiancheng Bioengineering Institute, Nanjing, China), superoxide dismutase (SOD) and malondialdehyde (MDA) (BioVision Incorporated, CA, U.S.A.) levels in colonic tissues were determined using commercial kits. Briefly, to determine the MDA level, colon tissue was homogenized in MDA lysis solution and centrifuged at 1300 × ***g*** for 10 min. We collected the supernatant, and MDA standards were prepared with ddH_2_O. Subsequently, thiobarbituric acid (TBA) was added to all standards and samples. The standards and samples were incubated at 95°C for 1 h and then cooled in an ice bath for 10 min. Analysis was carried out using 96-well microtiter plates, and the results were expressed in nmol/mg protein. SOD is one of the most important antioxidant enzymes. SOD catalyzes the transformation of superoxide anion into molecular oxygen and hydrogen peroxide and inhibits the reduction of the superoxide anion, which exhibits a linear relationship with the activity of xanthine oxidase (XO). The results were expressed as the % inhibition rate. The colorimetric method was used to measure the MPO level according to the theory that one unit of MPO activity degrades 1 μmol of hydrogen peroxide per minute at room temperature. The results were expressed as U/g tissue. Through a reaction with 5,5-dithiobis-(2-nitrobenzoic acid) (DTNB), we measured the GSH concentration. The absorbance was measured at 412 nm, and the GSH level was expressed as μmol/g tissue. The NO level was also measured using the colorimetric method. The absorbance was measured at 550 nm, and the results were expressed as µmol/g tissue.

### Data analysis

Statistical analysis was conducted with SPSS 20.0 software. The data were presented as the mean ± S.D. Differences between experimental groups were evaluated using one-way ANOVA followed by Dunnett’s test. A *P*-value less than 0.05 was considered statistically significant.

## Results

### Effect of OMT on the symptoms of DSS-induced intestinal inflammation in mice

**Figure 1:**
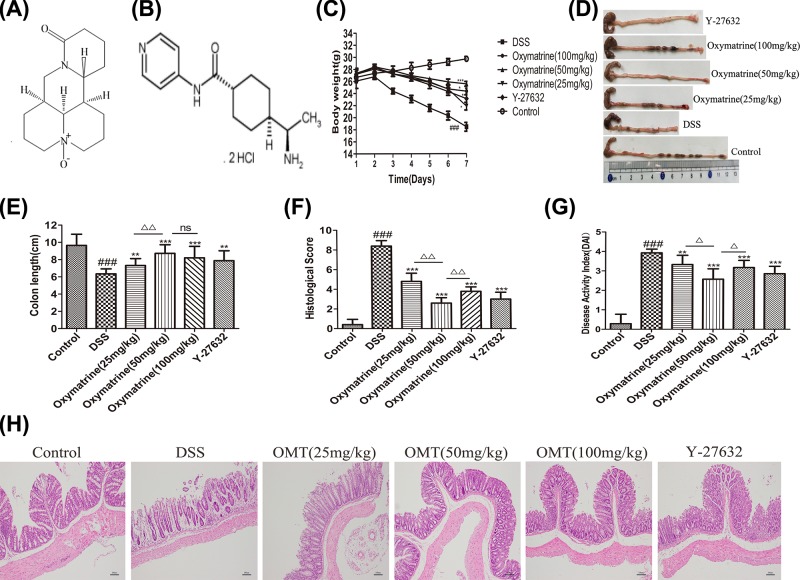
OMT attenuated the symptoms of DSS-i nduced intestinal inflammation in Mice (**A**) and (**B**) Molecular formula of OMT and Y-27632. (**C**) Mice were administered 3% DSS in drinking water throughout the 7-day experimental period. Body weight were recorded daily. (**D**) and (**E**) Macroscopic observation and assessment of colon length. (**F**) Histological scores were analyzed. (**G**) DAI of mice in each group. (**H**) Representative H&E-stained colon sections (magnification, scale bar = 100 μm). Values were expressed as the mean ± S.D., *n*=8 in each group. ^###^*P*<0.001, vs control group. **P<0.01, ***P<0.001, vs DSS group. ^Δ^*P*<0.05, ^ΔΔ^*P*<0.01, vs medium-dose oxymatrine group.

C_15_H_24_N_2_O_2_ and C_14_H_21_N_3_O·2HCl are the molecular formula of OMT and Y-27632 respectively, and their structure is shown in Figure 1A,B. After 7 days of treatment, severe intestinal inflammation was present in the DSS group, as evidenced by significant weight loss, colonic shortening and increased DAI and histologic scores; OMT treatment at doses of 100, 50 and 25 mg/kg and Y-27632 (10 mg/kg) limited body weight loss, diarrhea, bloody stools and colon length shortening. The normal control group exhibited stable weight gain without bloody stool, diarrhea, colon bleeding or colon shortening. Histopathological analysis showed that OMT and Y-27632 reduced colonic mucosal erosion and inflammatory cell infiltration, while the DSS group showed significant mucosal damage, ulceration, glandular destruction and inflammatory cell infiltration (Figure 1C-H). The DAI scores in the low-dose group and the high-dose group were significantly higher than those in the medium-dose OMT group. The colon length in the low-dose group was significantly shorter than that in the medium-dose group, while the colon length in the high-dose group was not significantly different from that in the medium-dose group. Taken together, these results demonstrated that OMT could exert protective effects against acute intestinal inflammation.

### Effect of OMT on expression of TJ proteins

**Figure 2:**
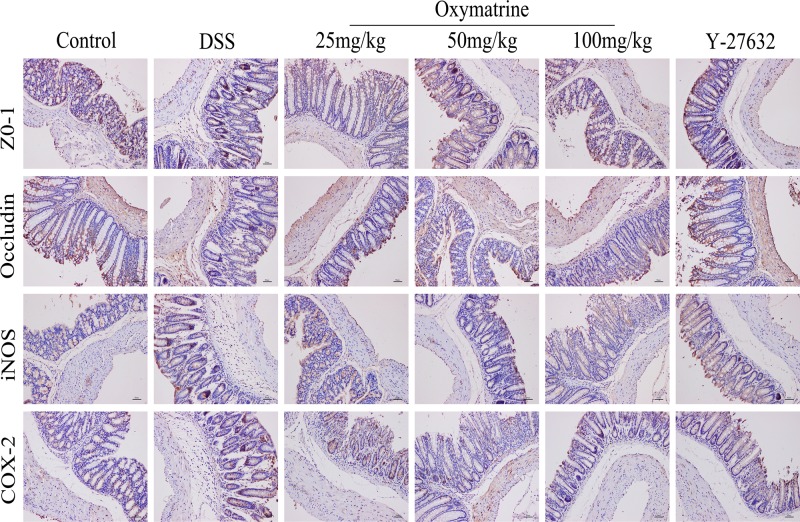
Effects of OMT on the levels of inflammatory factors and TJ proteins in colon Expressions of ZO-1, Occludin, iNOS and COX-2 in colon sections were assessed by IHC (magnification, scale bar = 50 μm).

**Figure 3:**
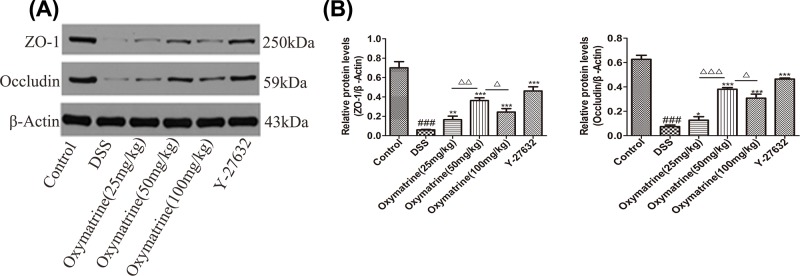
Effect of OMT on expression of TJ proteins (**A**) The colonic protein levels of ZO-1, Occludin were detected by Western blot. (**B**) Relative ratio levels were determined by densitometric analysis normalized to β-actin. Values represent means ± S.D. (*n*=3). ^###^*P*<0.001, vs control group. *P<0.05, **P<0.01, ***P<0.001, vs DSS group. ^Δ^*P*<0.05, ^ΔΔ^*P*<0.01, ^ΔΔΔ^*P*<0.001, vs medium-dose oxymatrine group.

TJs are closely related to the effectiveness and stability of the epithelial barrier and composed of a variety of proteins, including transmembrane proteins such as occludin, claudin and junctional adhesion molecules, as well as peripheral membrane proteins such as ZO-1, -2, -3. The interaction between occludin and ZO-1 plays an important role in maintaining TJ structure and epithelial barrier integrity [30–33]. Western blot analysis was used to determine the expression levels of occludin and ZO-1 to observe alterations in the TJ proteins. As shown in Figures 2 and 3A,B, compared with those in the control group, the expression levels of ZO-1 and occludin were markedly lower in the DSS group, whereas OMT and Y-27632 treatment at all doses dramatically elevated the levels of these proteins. Compared with those in the middle-dose OMT group, the ZO-1 and occludin protein levels in the low-dose group and the high-dose group were significantly lower. These data suggested that OMT could effectively protect the integrity of the epithelial barrier.

### Effect of OMT on oxidative stress

**Figure 4:**
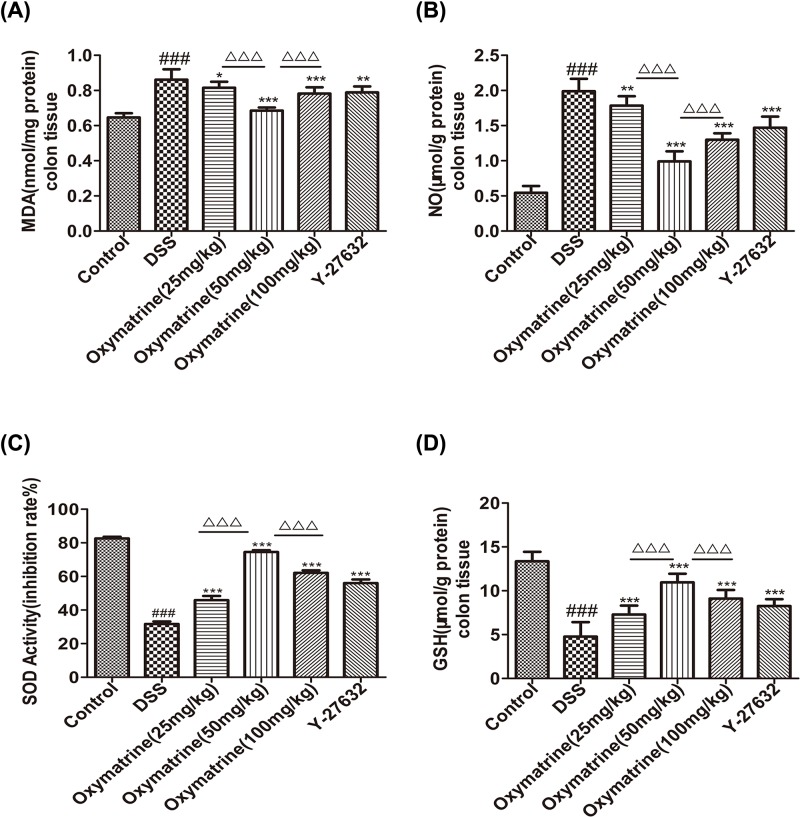
Effect of OMT treatment on oxidative stress (**A**),(**B**),(**C**) and (**D**) Biochemical analysis of MDA, NO, SOD and GSH in colon. Values were the mean± SD(*n*=8). ^###^*P*<0.001, vs control group. *P<0.05, **P<0.01, ***P<0.001, vs DSS group. ^ΔΔΔ^*P*<0.001, vs medium-dose oxymatrine group.

Increasing evidence has shown that oxidative stress induced by overproduction of reactive oxygen metabolites (ROMs) plays an important role in intestinal tissue damage in UC models [34]. As shown in Figure 4, MDA and NO levels were found to be greatly elevated in DSS-induced mice compared with those in control mice. In contrast, treatment of mice with DSS-induced acute intestinal inflammation with OMT and Y-27632 resulted in significant decreases in MDA and NO levels. The levels of antioxidants and antioxidant enzymes, such as GSH and SOD, in the colonic mucosa were examined in the control and experimental groups. The activities of antioxidants and antioxidant enzymes were found to be much lower in the mice with DSS-induced acute intestinal inflammation than in the control mice. The mice with DSS-induced acute intestinal inflammation that were treated with OMT and Y-27632 exhibited significantly increased antioxidant activity compared with that of the untreated DSS-induced acute intestinal inflammation mice. Compared with the middle-dose OMT group, the SOD and GSH levels in the low-dose group and the high-dose group were significantly reduced, while the MDA and NO levels were significantly increased. Taken together, these findings indicated that OMT treatment attenuated oxidative perturbations and promoted antioxidant defenses in the colon during the pathological IBD process.

### Effect of OMT on the secretion of inflammatory factors by damaged colonic tissue

**Figure 5:**
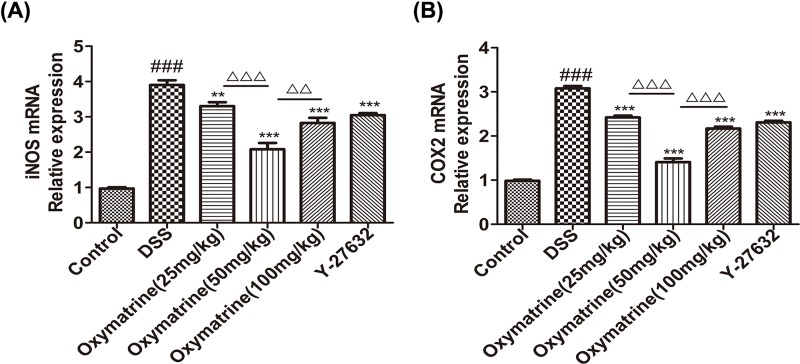
Effect of OMT on the mRNA levels of iNOS and COX-2 (**A**) and (**B**) Assessment of the mRNA expression levels of iNOS, COX-2 in colon. Values were the mean± S.D. (*n*=3). ^###^*P*<0.001, vs control group. **P<0.01, ***P<0.001, vs DSS group. ^ΔΔ^*P*<0.01, ^ΔΔΔ^*P*<0.001, vs medium-dose oxymatrine group.

RT-PCR were used to evaluate the impact of OMT on the expression levels of iNOS and COX-2 in colonic tissues. DSS was found to significantly induce iNOS and COX-2 expression in the mouse colon. The expression levels of the above pro-inflammatory factors in the DSS-induced acute intestinal inflammation mice were greatly reduced by OMT and Y-27632 treatment. Compared with those in the middle-dose OMT group, the levels of iNOS and COX-2 in the low-dose group and the high-dose group were significantly increased (Figure 5). These findings were consistent with the IHC findings, indicating that OMT exerted a potent anti-inflammatory effect in the DSS-induced acute intestinal inflammation mouse model.

### Effect of OMT on MPO activity

**Figure 6:**
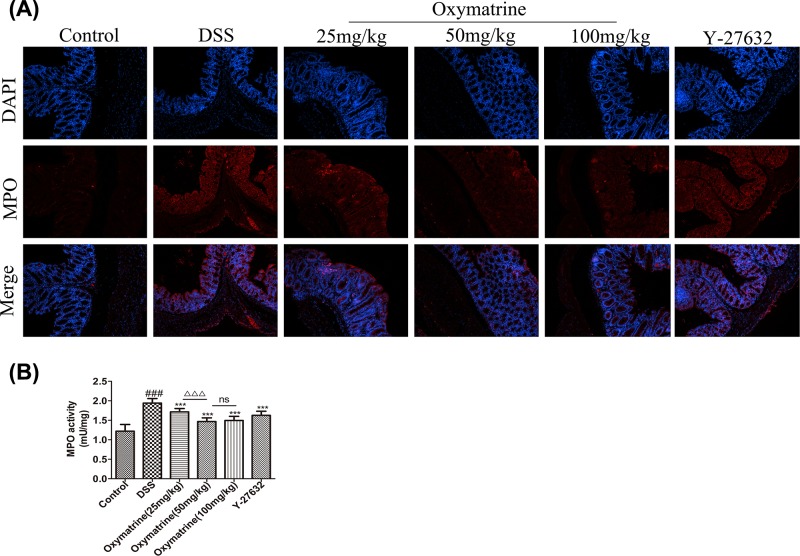
OMT inhibited MPO expression in DSS-induced intestinal inflammation mice (**A**) The result of immunofluorescence analysis of MPO (magnification, ×200). (**B**) Biochemical analysis of MPO. Values were the mean± S.D. (*n*=8). ^###^*P*<0.001, vs control group. *** *P*<0.001, vs DSS group. ^ΔΔΔ^*P*<0.001, vs medium-dose oxymatrine group.

Figure 6A showed the results of immunofluorescence analysis of MPO expression. MPO is an enzyme present in neutrophils, and the MPO concentration in macrophages and monocytes is considerably lower. MPO activity is recognized as a biochemical agent of inflammation in given tissues, and its expression is linearly correlated with neutrophil infiltration. The present study found that compared with the control group MPO expression was elevated in DSS-induced acute intestinal inflammation mice. On the contrary, the expression levels of MPO in mice were reduced after treatment with OMT and Y-27632. These findings were consistent with the trend of MPO expression determined by biochemical assays (Figure 6B), suggesting that OMT effectively controlled neutrophil infiltration during DSS treatment.

### Effects of OMT on the expression of ROCK1, ROCK2 and downstream molecular targets of the RhoA/ROCK signaling pathway

**Figure 7:**
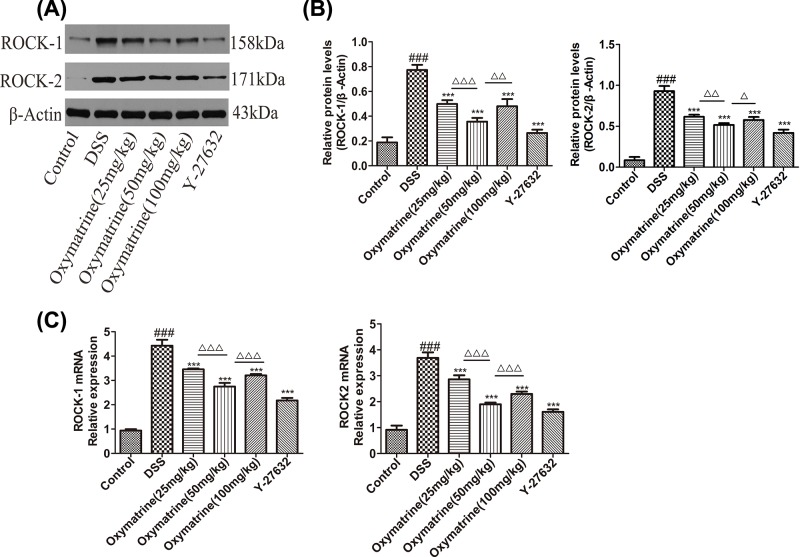
Effect of OMT on ROCK1 and ROCK2 in impaired colon (**A**) The colonic protein levels of ROCK1 and ROCK2 were detected by Western blot. (**B**) Relative ratio levels were determined by densitometric analysis normalized to β-actin. (**C**) The mRNA expression levels of ROCK-1 and ROCK-2 in colon were quantified. Values represent means ± S.D. (*n*=5). ^###^*P*<0.001, vs control group. **P<0.01, ***P<0.001, vs DSS group. ^Δ^*P*<0.05, ^ΔΔ^*P*<0.01, ^ΔΔΔ^*P*<0.001, vs medium-dose oxymatrine group.

**Figure 8:**
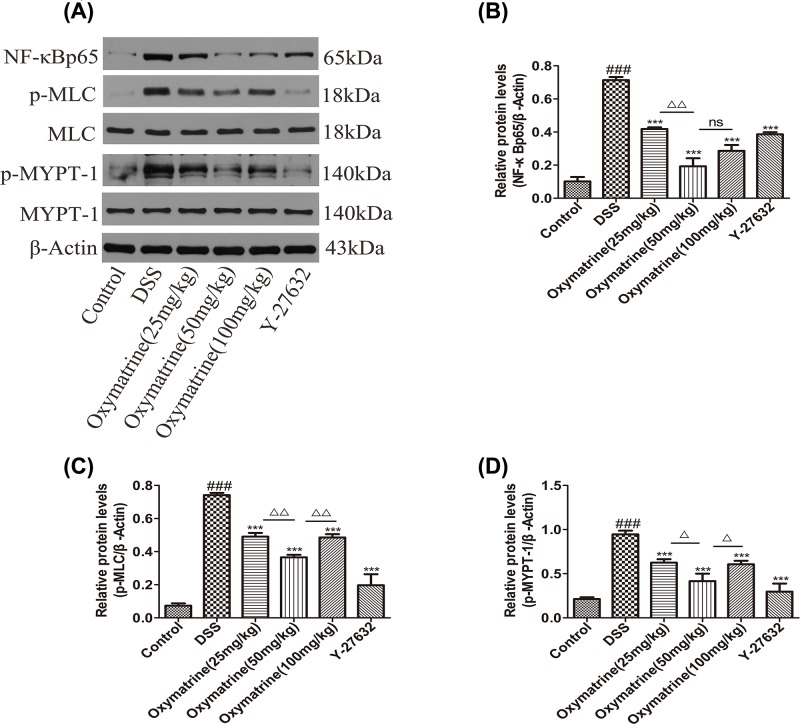
Effect of OMT on downstream molecular of ROCK signal pathway (**A**) The colonic protein levels of NF-κB, p-MLC, MLC, p-MYPT-1 and MYPT-1 were detected by Western blot. (**B**), (**C**) and (**D**) Western blot analysis of NF-κB, p-MLC and p-MYPT-1 expressions in colon. Values represent means ± S.D. (*n*=3). ^###^*P*<0.001, vs control group. *** *P*<0.001, vs DSS group. ^Δ^*P*<0.05, ^ΔΔ^*P*<0.01, vs medium-dose oxymatrine group.

The Rho kinase pathway regulates the production of inflammatory mediators [35]. ROCK1 and ROCK2 protein and mRNA levels increased during DSS administration in the colon tissue but decreased significantly after treatment with OMT and Y-27632 (Figure 7). As NF-κB, MLC and MYPT-1 are the key downstream molecules of the ROCK signaling pathway, Western blotting analysis of their levels was performed. As shown in Figure 8, the phosphorylation of MLC, MYPT-1 and NF- κB(p65) in the model group was significantly increased. However, treatment with Y-27632 and OMT down-regulated the expression of ROCK2, ROCK1, p-MLC (Ser19), p-MYPT1 (Thr696) and NF-κB(p65) substantially. Compared with those in the middle-dose OMT group, the ROCK-1 and ROCK-2 protein and mRNA levels in the low-dose group and the high-dose group were significantly increased, and the MLC and MYPT-1 phosphorylation levels were also significantly increased. The NF-κB phosphorylation level was significantly higher in the low-dose group than in the middle-dose group, while the high-dose group showed no significant difference in NF-κB phosphorylation levels compared with those in the middle-dose group. Nevertheless, there were no significant differences in the total amounts of MYPT-1 and MLC in each group (data not shown). These results showed that OMT could effectively inhibit the expression and activation of ROCK.

### Effects of OMT on the differentiation of Th17 and Treg cells

**Figure 9:**
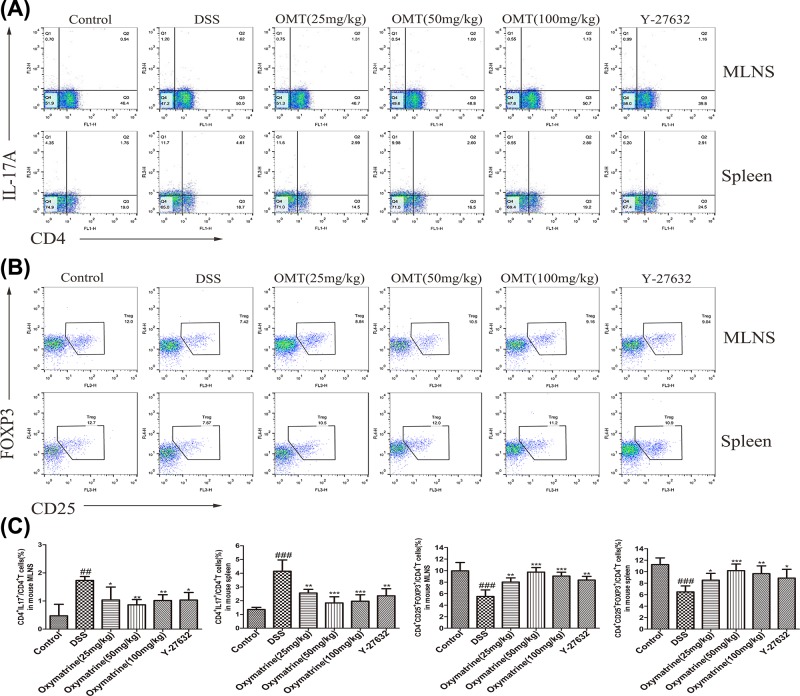
Effect of OMT on the differentiation of Th17 and Treg cells (**A**) and (**B**) The differentiation of Th17 and Treg cells in MLNs and spleen of mice was analyzed by flow cytometry. (**C**) The average percentage of Th17 and Treg cells in MLNs and spleen. Values represent means ± S.D. (*n*≥3). ^##^*P*<0.01, ^###^*P*<0.001, vs control group. *P<0.05, **P<0.01, ***P<0.001, vs DSS group.

**Figure 10:**
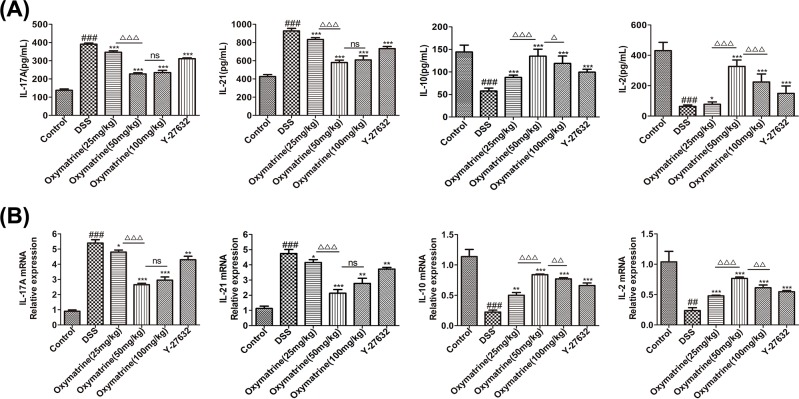
Effect of OMT on the levels of cytokine related to Th17 and Treg cells (**A**) ELISA analysis of IL-17A, IL-21, IL-10 and IL-2 in colon. Values represent means ± S.D. (*n*=8). (**B**) The mRNA expression levels of IL-17A, IL-21, IL-10 and IL-2 in colon were quantified. Values represent means ± S.D. (*n*=3). ^###^*P*<0.001, vs control group. *P<0.05, **P<0.01, ***P<0.001, vs DSS group. ^ΔΔ^*P*<0.01, ^ΔΔΔ^*P*<0.001 vs medium-dose oxymatrine group.

**Figure 11:**
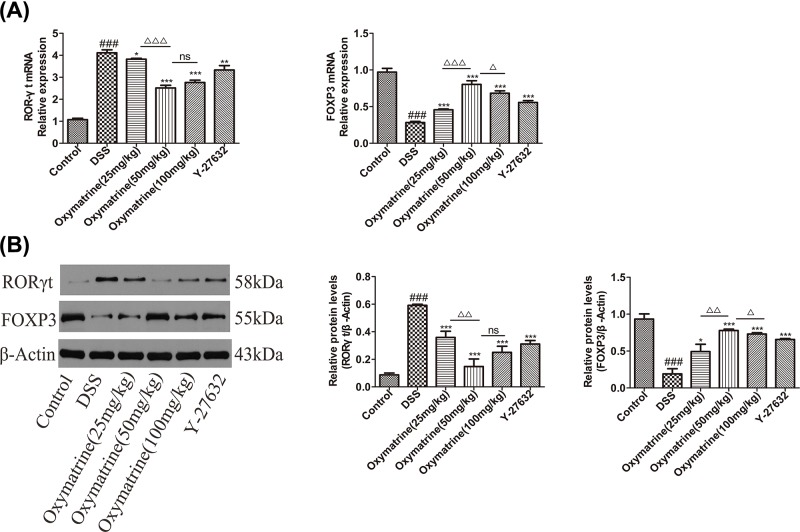
Effect of OMT on the transcription factors of Th17 and Treg cells (**A**) The mRNA expressions of ROR-γt and Foxp3 were determined by qRT-PCR. (**B**) Western blot analysis of ROR-γt and Foxp3 expressions in colon. Values represent means ± S.D. (*n*=3). ^###^*P*<0.001, vs control group. *P<0.05, **P<0.01, ***P<0.001, vs DSS group. ^Δ^*P*<0.05, ^ΔΔ^*P*<0.01, ^ΔΔΔ^*P*<0.001, vs medium-dose oxymatrine group.

To determine the anti-inflammatory effects of OMT *in vivo*, the balance between Th17 and Treg cells in the spleen and MLNs was analyzed. We found that both the MLNs and spleens in the DSS treatment group showed increased Treg percentages and decreased Th17 cell percentages (Figure 9). In addition, we measured characteristic cytokine levels that have been recognized as substantially important elements for the differentiation of Th17 and Treg subgroups in colonic tissue. Compared with DSS group, the levels of IL-17A and IL-21, which are important cytokines secreted by Th17 cells, were down-regulated in the OMT and Y-27632 treatment groups, while those of IL-2 and IL-10, which are important to the regulation of the differentiation of Treg cells, were up-regulated in the OMT and Y-27632 treatment groups, as determined by ELISA and RT-PCR analysis. (Figure 10). To examine the molecular mechanism underlying the effects of OMT on the Th17 and Treg subsets, the expression of nuclear transcription elements in these subsets was determined through Western blotting and RT-PCR. In contrast to the DSS group, OMT and Y-27632 treatment reduced the expression of RORγt but enhanced the expression of FOXP3 at both the mRNA and protein levels (Figure 11). Compared with those in the medium-dose OMT group, the levels of IL-17A, IL-21 and RORγt in the low-dose group were significantly increased, while the levels of IL-17A, IL-21 and RORγt in the high-dose group were not significantly altered. The levels of IL-10, IL-2 and FOXP3 in the low-dose group and the high-dose group were significantly lower than those in the middle-dose group. There were no significant differences in the ratio of CD4^+^IL-17A^+^T cells to CD4^+^CD25^+^FOXP3^+^T cells between the low-dose or high-dose group and the middle-dose group. Collectively, these results indicated that the ability of OMT to ameliorate DSS-induced acute intestinal inflammation was associated with the inhibition of Th17 cell differentiation and the promotion of Treg cell differentiation.

## Discussion

IBD is a class of chronic gastrointestinal diseases that is caused by multiple factors and largely consists of two main types: CD and UC. Many people suffer from IBD worldwide [36]. Intestinal mucosal barrier damage [1], abnormal overproduction of pro-inflammatory cytokines in inflammatory mucosa [37], oxidative stress and immune dysfunction are key factors in the pathogenesis of UC [36,38–39]. Thus, we sought to determine whether OMT suppressed inflammation in the colon by attenuating IEB dysfunction, modulating pro-inflammatory cytokines, relieving oxidative stress and restoring the balance between Th17 and Treg cells.

In IBD, epithelial-extrinsic mediators, such as pro-inflammatory cytokines derived from activated innate immune cells, are effective inducers of IEC apoptosis and pathological cell shedding and subsequently impair epithelial barrier function [3,40–41]. It is of great importance to ensure that the IEB is maintained because it can prevent not only toxin penetration but also electrolyte loss [32]. TJs are an important component of the IEB and are composed of a variety of proteins, including intracellular proteins and transmembrane proteins, such as ZO-1 and occludin [31,32]. As a downstream effector of RhoA, ROCK has been demonstrated to play an essential role in regulating biological pathways, such as those that impact smooth muscle tension levels and those that impact different kinds of physiological characteristics related to actin cytoskeletal changes, including migration, cell adhesion, contraction and motility [42,43]. In autoimmune diseases, including UC, the differentiation of Th17 can be induced by ROCKs, and the Th17/Treg balance can also be influenced by ROCKs [24]. Given that RhoA/ROCK has extensive regulatory abilities, its roles in UC and other autoimmune and inflammatory diseases have previously been reported. Therefore, we hypothesized that RhoA/ROCK may be involved in the pathogenesis of UC. Our findings showed that OMT significantly up-regulated the expression of ZO-1 and occludin while down-regulated the expression of ROCK1 and ROCK2 and inhibited the phosphorylation of MLC, MYPT-1 and NF-κBp65. In addition, OMT could also regulate inflammation and affect the balance between Th17 and Treg cells. In summary, these findings indicated that OMT may serve as a promising and effective treatment for UC. Its therapeutic effects create various transcriptional pathways, including MLC and NF-κB.

Oxidative stress is another important factor that causes UC. Oxidative stress has been suggested to be associated with the activation, infiltration and recruitment of neutrophils in the inflammatory colonic mucosa during acute inflammation, as well as the destruction of cellular macromolecules, such as DNA, lipids and proteins. The free radical chain reaction is enhanced, the integrity of the intestinal mucosal barrier is disrupted, and inflammatory mediators are activated due to lipid peroxidation and oxidative stress [44–46]. As a result, the MDA level is elevated in the colon. Therefore, MDA is generally used as a marker of free radical-induced lipid peroxidation and is indicative of oxidative damage. The intestinal mucosa has a complex antioxidant system to maintain cellular redox equilibrium and counteract oxidative stress. SOD is an enzymatic scavenger that converts the superoxide anion into hydroperoxides [45–47]. Previous studies have shown that GSH protects normal tissues and cells from oxidative damage, reduces the number of protein sulfhydryl groups (-SH) and prevents -SH groups from reacting with free radicals. NO is a potent pro-inflammatory mediator. NO is primarily produced in specific cell types (including smooth muscle cells and macrophages) by iNOS upon stimulation with bacterial endotoxins and inflammatory cytokines, such as TNF-α. iNOS overexpression in the mucosa (such as the mucosa of the gastrointestinal tract) is related to the development of inflammatory diseases, including IBD. In inflammatory diseases, infiltrating cells such as macrophages produce high levels of NO. NO promotes the infiltration of neutrophils into the midgut and distal colon, resulting in tissue damage. Neutrophils, lymphocytes and colonic epithelial cells have been reported to be directly related to local tissue damage and disease progression in IBD patients [45]. In the present study, the levels of GSH and SOD in the control group were much higher than those in the DSS group. OMT at different doses could reduce MDA and NO activity in DSS-induced colitis while increasing SOD and GSH activity, which suggested that OMT may serve as a major defense against oxidative stress. These results indicated that OMT successfully inhibited DSS-induced oxidative stress in acute intestinal inflammation and improved the enzymatic defense system, thereby maintaining the cellular antioxidant/oxidation balance.

In addition, OMT treatment successfully reduced the levels of proi-nflammatory chemokines and cytokines. Increased levels of different chemokines and cytokines promote the development of IBD. Cytokines and chemokines make up a complex network that can regulate the immune process during intestinal inflammation. COX-2 is an important rate-limiting enzyme in arachidonic acid metabolism and is an inducible cyclooxygenase isoenzyme. According to previous clinical studies, iNOS induces COX-2 expression during inflammation, and COX-2 in turn promotes iNOS expression. iNOS and COX-2 are recognized as two key enzymes in the development and transformation of colitis lesions [48]. The present study showed that intraperitoneal injection of OMT significantly reduced the overexpression of iNOS and COX-2, indicating that the protective function of OMT was related to OMT-mediated inhibition of the activation of inflammatory factors.

MPO, an enzyme in neutrophils, has been shown to be an effective marker for assessing granulocyte infiltration in colon tissue after the induction of colitis. Once released, MPO catalyzes the formation of ROS, which are associated with the pathogenesis of IBD [49]. In the present study, intraperitoneal injection with OMT effectively inhibited DSS-induced MPO activity in the mouse colon, indicating that OMT effectively reduced infiltration of neutrophils.

The main clinical symptoms of acute intestinal inflammation caused by 3% DSS treatment for 7 days were weight loss, diarrhea, bloody stools, crypt distortion, tissue edema, glandular damage, reduced colonic length and inflammatory cell infiltration. The length of colons induced by inflammation, macroscopically visible damage and DAI scores were improved noticeably upon treatment with medium and high doses of OMT. In general, these data suggested that OMT could relieve the acute intestinal inflammation induced by DSS via blockade of the RhoA/ROCK signaling pathway.

Multiple indicators in our study showed no statistically significant differences between the middle-dose and high-dose groups. One possible explanation is that there might have been individual differences between the mice, as the mice were free to drink the DSS, and we could not guarantee a specific dose in each group of mice, which may have led to deviations between groups. Another possibility is that the sample size was insufficient. The optimal dose of OMT for UC is still unclear and the effect may be lower or higher than that observed. However, this possibility does not affect the conclusion that OMT can significantly alleviate DSS-induced acute intestinal inflammation by acting on RhoA/ROCK signaling. We also found that OMT alleviated the symptoms of UC more significantly than Y-27632. This effect may be due to multiple pharmacological targets of OMT or may be due to the side effects of Y-27632 in animals.

## Conclusions

In summary, the present study demonstrated that OMT could exert its potential therapeutic effects on DSS-induced acute intestinal inflammation in the following aspects: alleviating symptoms, protecting the integrity of the epithelial barrier, suppressing inflammation, relieving oxidative stress and maintaining the balance between Th17 and Treg cells. Our study provided additional information regarding the important role that the RhoA/ROCK pathway played in the pathogenesis of UC. Importantly, inhibiting the RhoA/ROCK pathway might be a new approach that can be used in UC therapy. All of these findings indicated that OMT may serve as a potential treatment for UC.

## Abbreviations

CD: Crohn’s disease
DAB: diaminobenzidine
DAI: disease activity index
DSS: dextran sodium sulfate
ELISA: enzyme-linked immunosorbent assay
GSH: glutathione
IBD: inflammatory bowel disease
MDA: malondialdehyde
MLC: myosin light chain
MLCP: MLC phosphatase
MLN: mesenteric lymph node
MPO: myeloperoxidase
MYPT-1: myosin phosphatase-targeting subunit-1
OMT: oxymatrine
qRT-PCR: quantitative real-time PCR
ROCK: Rho kinase
ROS: reactive oxygen species
SOD: superoxide dismutase
TJ: tight junction
UC: ulcerative colitis
ZO: zonula occluden
