# Expression of Concern: Protective effects of oxymatrine against DSS-induced acute intestinal inflammation in mice via blocking the RhoA/ROCK signaling pathway

**DOI:** 10.1042/BSR20182297_EOC

**Published:** 2026-08-25

**Authors:** Yifan Wang, Zhexing Shou, Heng Fan, Meng Xu, Qianyun Chen, Qing Tang, Xingxing Liu, Hui Wu, Man Zhang, Ting Yu, Shuangjiao Deng, Yujin Liu

**Affiliations:** Department of Integrated Traditional Chinese and Western Medicine, Union Hospital, Tongji Medical College, Huazhong University of Science and Technology, Wuhan 430022, China

**Keywords:** immunity, intestinal epithelial barrier, oxidative stress, Oxymatrine, RhoA/ROCK pathway, ulcerative colitis

*Bioscience Reports* has been made aware of potential issues surrounding the scientific validity of this paper and is hence issuing an Expression of Concern to notify readers.

A reader has notified us of a partial duplication that occurs within Figure 2, between a region of the Occludin/control panel and a region of the Occludin/Y-27632 panel. The authors provided raw data and a replacement image; however, the raw data contained the same region of similarity as seen in the Figure panels.

As such, concerns remain. The article will therefore remain published but with an associated Expression of Concern.

